# Amendments to Be Moved by Colonel Raw on Behalf of the College of Nursing, Limited

**Published:** 1919-04-12

**Authors:** 


### NURSES' REGISTRATION BILL.

### Amendments to be moved by Colonel Raw on behalf of the College of Nursing, Limited.

REPRESENTATION ON GENERAL NURSING COUNCIL.

For Clause 4 (1) (g) substitute the following:—

"Two-thirds of the Council shall be elected by the nurses on the Woman Nurses' General Register under this Act; and of the persons so elected four-sixths shall represent England and Wales, one-sixth Scotland, and one-sixth Ireland. Of the persons elected by the nurses on the General Register to represent England, and Wales. Scotland and Ireland respectively, five-sixths shall be nurses on the Woman Nurses' General Register."

PROVISIONAL NURSING COUNCIL.

In Clause 4 (1) (k) to omit all the words following "nurse" in line 11, and to substitute the following:—

"Provided that on the first constitution of the Council there shall be thirty-six persons, appointed as follows:—

Three by the Council of the British Medical Association.

Three by the Council of the Medico-Psychological Association.

Three by the Council of the Association of Poor Law Unions.

Three by the Council of the British Hospitals Association.

Twelve by the Central Committee for the State Registration of Nurses.

Twelve by the Council of the College of Nursing.

And the persons so appointed shall hold office until they have framed rules regulating the admission to the Register of persons already in practice as trained nurses at the commencement of this Act and until the Register of persons so entitled to be registered contains the names of not fewer than 20,000 persons; whereupon an election of direct representatives of registered nurses shall be held and the persons appointed as above shall cease to hold office."

TERM OF OFFICE.

In Clause 7 to omit the words "At the expiration of two years from the date of the first constitution of the Council," and to substitute:—

"At the expiration of three years from the date of the first election of direct representatives of registered nurses on the Council."

DUTIES AND POWERS OF COUNCIL.

In Clause 11 omit the last section: "And provided—with this Act." (Page 7, lines 24-28 inclusive.)

PROVISION FOR EXISTING NURSES.

In Clause 12 insert before 12 (1) the words:—

"Is a registered nurse member of the College of Nursing, Ltd."

WHO MAY BE REGISTERED.

In Clause 13, line 5, insert the word "general" before "training."

APPEAL TO THE PRIVY COUNCIL WITH REGARD TO RECOGNITION OF HOSPITALS.

In Clause 14, move the omission of this clause.

FEES AND EXPENSES.

In Clause 17 (1) add:—

"Provided that such fee shall not exceed £1 for registration."

NO AUTHORITY TO PRACTISE MEDICINE.

In Clause 25 omit all words after "midwifery."

